# Irritable Bowel Syndrome and Gluten-Related Disorders

**DOI:** 10.3390/nu12041117

**Published:** 2020-04-17

**Authors:** Paolo Usai-Satta, Gabrio Bassotti, Massimo Bellini, Francesco Oppia, Mariantonia Lai, Francesco Cabras

**Affiliations:** 1 Gastroenterology Unit, Brotzu Hospital, 09121 Cagliari, Italy; f.oppia@tiscali.it (F.O.); francescocabras@aob.it (F.C.); 2 Gastroenterology & Hepatology Section, Department of Medicine, University of Perugia, 06156 Perugia, Italy; gabassot@tin.it; 3 Gastrointestinal Unit, Department of Translational Research and New Technologies in Medicine and Surgery, University of Pisa, 56010 Pisa, Italy; mbellini58@gmail.com; 4 Gastroenterology Unit, University of Cagliari, 09042 Monserrato, Italy; marlai@aoucagliari.it

**Keywords:** irritable bowel syndrome, celiac disease, nonceliac gluten/wheat sensitivity, gluten-free diet

## Abstract

**Background**: Irritable bowel syndrome (IBS) is frequently associated with celiac disease (CD) and nonceliac gluten/wheat sensitivity (NCGS/NCWS), but epidemiological and pathophysiological aspects are still unclear. Furthermore, a gluten-free diet (GFD) can positively influence IBS symptoms. **Methods**: A comprehensive online search for IBS related to CD, NCGS and GFD was made using the Pubmed, Medline and Cochrane databases. **Results**: Although a systematic screening for CD in IBS is not recommended, CD prevalence can be increased in diarrhea-predominant IBS patients. On the other hand, IBS symptoms can be persistent in treated CD patients, and their prevalence tends to decrease on a GFD. IBS symptoms may overlap and be similar to those associated to nonceliac gluten and/or wheat sensitivity. Increased gut permeability could explain the gluten/wheat effects in IBS patients. Finally, a GFD could improve symptoms in a subgroup of IBS patients. **Conclusions**: The possible interplay between IBS and gluten-related disorders represents a scientifically and clinically challenging issue. Further studies are needed to confirm these data and better clarify the involved pathophysiological mechanisms.

## 1. Introduction

Irritable bowel syndrome (IBS) is the most frequently diagnosed functional gastrointestinal disorder, causing abdominal pain, bloating, diarrhea and constipation [1]. This condition affects 10–15% of the general population and is associated with a decreased quality of life (QoL). IBS is classified into three main subtypes according to the predominant bowel habit: constipation-predominant (IBS-C), diarrhea-predominant (IBS-D) and mixed bowel habits (IBS-M) [2,3,4,5,6]. Since there are no available biological markers that clearly identify such patients, the diagnosis of IBS is usually made based on the symptoms according to the Rome IV criteria [7]. These criteria suggest performing limited laboratory studies, including serological tests for celiac disease (CD) in patients with IBS-D and IBS-M. The initial treatment is directed towards lifestyle and, eventually, dietary modification. Subsequently, an appropriate pharmacotherapy can be proposed [8]. 

Although a mutual relationship between CD and IBS has been hypothesized, the available evidence is controversial [9,10]. In addition, the symptom complex of IBS-D may overlap and resemble that associated with nonceliac gluten/wheat sensitivity (NCGS/NCWS) [11]. Finally, a gluten-free diet (GFD) has been proposed in a subgroup of patients with IBS as a possible therapeutic option [12]. This review aimed to evaluate and clarify the relationship between IBS and gluten-related disorders, including the impact of a GFD in IBS patients. 

## 2. Materials and Methods

We performed a comprehensive online search of Medline, Cochrane and the Science Citation Index using the keywords “irritable bowel syndrome”, “celiac disease”, “non celiac gluten sensitivity” and “gluten free diet” in various combinations with the Boolean operators *and*, *or*, and *not*, selecting articles published in English between January 2000 and December 2019. 

## 3. IBS and CD

CD is a chronic, gluten-related disorder characterized by small intestinal mucosal inflammation and malabsorption in genetically predisposed individuals. The prevalence of CD in the worldwide general population is reported to be about 1% [13,14]. The clinical picture of CD often overlaps with that of IBS, and several studies suggest that IBS patients are at increased risk of CD [9,15]. In contrast to IBS, symptoms may resolve if the disease is recognized and a strict GFD is respected. However, sufficient data are not available to demonstrate a higher prevalence of CD in patients with IBS-D compared with those with IBS-C or IBS-M. International guidelines yield conflicting recommendations about systematic screening for CD in IBS individuals [16,17,18]. From 2002 to 2007, the American Gastroenterological Association and the British Society of Gastroenterology suggested limited serological tests, whereas the American College of Gastroenterology did not recommend any laboratory investigations. A meta-analysis by Ford et al. [15] included 14 studies, comprising 4204 individuals. The prevalence of histology-proven CD in IBS patients was more than four-fold that in controls without IBS. A more recent meta-analysis [9] included 36 eligible studies, comprising 9275 subjects meeting the criteria for IBS. Pooled odds ratios (ORs) for positive antiendomysial antibodies (EMA) and/or tissue transglutaminase antibodies (tTG), and histology-proven CD in IBS subjects versus controls were 2.75 (95% CI 1.35–5.61), and 4.48 (95% CI 2.33–8.60). The prevalence of biopsy-positive CD was significantly higher across all subtypes of IBS. Also, the Rome IV foundation suggested that serologic tests for CD should be performed in patients with IBS-D and IBS-M who fail empiric therapy [7]. According to recent Canadian guidelines [6], testing for CD could be suggested in IBS-D rather than in IBS-C patients, although the studies concerning the role of celiac testing in IBS were of low-quality. On the other hand, studies from the United States [9,19], including a recent AGA technical review [5], did not identify an increase in the prevalence or in the ORs of CD in patients with IBS. In any case, universal screening for CD in every IBS patient is presently not recommended.

## 4. CD and IBS

Clinical practice suggests that many patients with CD have persistent digestive symptoms despite long-term GFD. Such (a) persistence of symptoms notwithstanding, strict dietary restrictions, is frustrating and may even lead to poor dietary adherence. More solid data on these clinical findings would be useful to improve the management and follow-up of celiac patients. 

Several studies have suggested that the prevalence of IBS symptoms among patients with CD on GFD may be higher than in the general population [20,21], but no conclusive data are available about the actual prevalence of functional gastrointenstinal disorders in patients with CD. In addition, the association with autoimmune diseases, microscopic colitis, or small intestinal bacterial overgrowth may be a further diagnostic confounding factor [13]. Barratt et al. [22] showed that IBS is more prevalent in CD on GFD in comparison with age-matched and sex-matched controls. The prevalence of IBS in CD was 22%. IBS additional symptoms were associated with reduced QoL and an increased likelihood of anxiety and depression. In 2013, a meta-analysis [10] showed a pooled prevalence of IBS symptoms of 38% (95% CI, 27.0–50.0%) in all patients with CD. Furthermore, celiac patients displayed a pooled OR for IBS symptoms that was higher than controls (5.60; 95% CI, 3.23–9.70). Improved adherence to a GFD might be associated with a reduction in symptoms. A more recent study [23] evaluated the prevalence and severity of IBS symptoms related to GFD in a group of CD patients. Based on a variable duration of GFD, patients were classified into short-term GFD (one to two years) and long-term GFD (greater than three years) groups and compared with a group of healthy controls. Although there were no differences in symptoms between the short- and long-term GFD groups, both had a worse symptom score than controls (*p* = 0.03 and *p* = 0.05, respectively). In another recent study [24] adult CD patients were studied at diagnosis, six months, and one year after GFD using Rome III criteria for IBS. At diagnosis and after one year of GFD, 52% and 22% of patients fulfilled the criteria for IBS, respectively. Therefore, IBS was persistent in treated CD patients, but its prevalence significantly decreased on a GFD.

## 5. IBS, Gluten, Wheat, and NCGS/NCWS

The presence of intestinal and extraintestinal symptoms related to gluten-containing food without the diagnostic findings of CD or wheat allergy has recently been named nonceliac gluten sensitivity (NCGS) [25]. Unlike CD, NCGS has no available specific diagnostic markers [26]. The complex of digestive symptoms associated with NCGS, such as diarrhea, bloating, or abdominal pain, may overlap and be similar to those caused by IBS-D [11]. The main difference between NCGS and IBS is usually based on the fact that patients with NCGS self-report symptoms when consuming gluten. Conversely, IBS patients generally do not report gluten ingestion as a specific stimulus for their symptoms [27]. However, food plays an important provocative role in IBS symptoms, and up to 80% of IBS patients complain of postprandial discomfort. Furthermore, many patients report presumed food intolerances [28,29]. According to recent evidence, the spectrum of symptoms that occur in NCGS patients may be due not only to gluten proteins, but also to other wheat-related components. Therefore, the term nonceliac wheat sensitivity (NCWS) has been coined [30,31]. Wheat contains a number of nongluten compounds that could produce digestive symptoms. Some of these compounds could be related to FODMAPS (fermentable oligo-, di-, and monosaccharides and polyols), specifically fructans [32]. The mechanism by which wheat or specific wheat components such as gluten cause IBS-type symptoms remains debatable [33]. In a study using confocal endomicroscopy, wheat administered endoscopically into the duodenal mucosa was able to affect the small intestinal mucosa integrity [34]. In a more recent study, intestinal permeability was significantly increased after gluten challenge in a group of gluten-sensitive, nonceliac IBS-D patients [35]. 

It can thus be hypothesized that an incomplete degradation of gluten and other wheat proteins allows undigested peptides to cross a more permeable mucosal barrier and provoke symptoms. This pathophysiological mechanism could be present at least in a subset of patients with IBS [36,37]. On the other hand, the incomplete knowledge of the pathogenesis and pathophysiology of IBS and NCGS/NCWS does not clarify whether these entities are separate, related, or overlap. Table 1 summarizes the most significant evidence on IBS related to gluten, wheat, and NCGS/NCWS.

## 6. IBS and Gluten/Wheat-Free Diet

Based on the above evidence, a gluten- (and also wheat-) free diet appears to represent a potential and appealing dietary intervention for a subset of patients with IBS [38]. There are several double-blind, placebo-controlled and randomized clinical trials evaluating the effect of GFD on IBS. Table 2 summarizes the most significant studies on this topic. 

A total of 60 IBS patients completed a double-blind randomized placebo-controlled study [41], in which the recruited subjects underwent GFD for four weeks, followed by a rechallenge of gluten-free bread or cereal-containing bread. This study showed that the gluten challenge group had higher symptom scores. A randomized clinical trial [39] was instead carried out in 45 patients with IBS-D, whose participants underwent either a four-week trial of a GFD or a gluten-containing diet. The authors demonstrated that daily bowel movements increased in patients assuming a gluten-containing diet. Another study [40] performed in 41 patients also showed a significant reduction in IBS-Symptom Severity Score (*p* < 0.001) in IBS-D patients after a six-week GFD. An Italian multicenter study [36] achieved a symptomatic improvement in 55 out 77 IBS patients (71.4%) after a three-week GFD, followed by a double-blind gluten challenge versus placebo, in which 18 out 53 responder patients with IBS (34%) had symptom relapse. Recently [42], a combination of low FODMAP diet and GFD (LFD-GFD) had positive effects in patients with CD and coexisting functional digestive symptoms. The authors observed a significant reduction in the VAS (visual analog scale) for abdominal pain in the LFD-GFD group versus the normal GFD group (*p* < 0.01). 

Concerning gluten as part of the wheat structure, wheat sensitivity has also been hypothesized in IBS patients. A large study [37] including 920 IBS patients with a self-reduced wheat diet performed an elimination diet for four weeks, followed by a double-blind, placebo-controlled challenge. The results showed that 30% of patients had NCWS, and were asymptomatic on an elimination diet. On the other hand, a double-blind placebo-controlled crossover trial [30] showed that participants with self-reported NCGS (and IBS symptoms) following a GFD reported further improved symptoms by LFD, and no specific effects of gluten were found. In a recent meta-analysis [12] including nine studies, GFD was associated with reduced global IBS symptoms compared with a control diet (RR = 0.42; 95% CI 0.11 to 1.55; I2 = 88%), although this was not statistically significant. The authors concluded that the available scientific evidence was not sufficient to recommend a GFD to improve IBS symptoms. According to the most recent evidence, recent Canadian guidelines [6] on the IBS management recommend against GFD in the treatment of IBS.

## 7. Conclusions

The mutual interplay between IBS and gluten-related disorders represents a topic of increasing interest. Although the prevalence of CD may be increased in IBS-D patients, universal screening for CD is not presently recommended in these patients. However, some evidence shows that in patients with CD on GFD, the persistence of digestive symptoms can be related to IBS. Moreover, the clinical picture of IBS can overlap with NCGS and NCWS, and an increased bowel permeability could explain the mechanism by which gluten and/or wheat can provoke symptoms in IBS subjects. Finally, GFD could decrease the impact of symptoms in a subset of IBS patients. Further studies are needed to assess the role of gluten-related disorders in IBS and vice versa.

## Figures and Tables

**Table 1:** Summary of the most significant studies on IBS related to gluten/wheat and NCGS/NCWS.

Authors (Ref)	Study Design	Study Method	Participants	Results
Potter [31]	Population-based study	Multivariate analysis	3115	NCWS was associated with IBS (OR: 3.55)
Fritscher–Ravens [34]	Prospective controlled study	Confocal endomicroscopy before and after wheat administration	36 IBS	IEL and intervillous spaces increased after wheat endoscopic challenge
Wu [35]	Double-blinded gluten challenge	Immuno-histochemistry by endoscopic biopsies	27 IBS-D	Increased gut permeability after gluten challenge
Elli [36]	Double-blinded trial	GFD and gluten challenge	134 with functional disorders (77 IBS)	14% of patients meet NCGS criteria
Carroccio [37]	Double-blinded trial	Wheat-free diet and wheat challenge	920 IBS	70 IBS with NCWS

Notes: IBS-D: irritable bowel syndrome with diarrhea, GFD: gluten-free diet, NCGS: nonceliac gluten sensitivity, NCWS: nonceliac wheat sensitivity, OR: odds ratio.

**Table 2:** Summary of the most significant studies on gluten and wheat-free diet in IBS.

Authors (Ref)	Study Design	Study Duration	Participants	Diet Methods	Results
Vasquez Roque [39]	RCT	6 months	45 IBS-D	GFD and gluten challenge	More bowel movements on gluten challenge
Aziz [40]	Prospective study	6 weeks	41 IBS-D	GFD	Symptoms improved on GFD
Zanwar [41]	DBP trial	4 weeks	60 IBS-D	GFD and gluten challenge	Symptoms worsened on gluten challenge
Elli [36]	DBP trial	3-week GFD, followed by 1-week gluten challenge	77 IBS	GFD and gluten challenge	Symptoms improved in 71% of IBS (34% relapsed on gluten challenge)
Roncoroni [42]	RCT	21 days	50 celiac patients with IBS symptoms	GFD-LFD	Better symptom impact in GFD-LFD than GFD alone
Biesiekierski [30]	DBP trial	2-week LFD, followed by 1 week low, high gluten or placebo	37 IBS-NCGS	High and low gluten challenge	No gluten effect on IBS symptoms; wheat sensitivity hypothesized
Carroccio [37]	DBP trial	5 weeks	276 IBS and wheat sensitivity	Wheat-free diet and wheat challenge	Asymptomatic on wheat-free diet and symptoms increased on wheat challenge
Dionne [12]	Meta-analysis	Variable	111 GFD 397 LFD	GFD and LFD	Low evidence on GFD in IBS

Notes: DBP: double-blinded placebo-controlled, RCT: randomized clinical trial, IBS-D: irritable bowel syndrome with diarrhea, GFD: gluten-free diet, LFD: low FODMAP diet; NCGS: nonceliac gluten sensitivity.
